# Plasma Metabolome Signature Indicative of *BRCA1* Germline Status Independent of Cancer Incidence

**DOI:** 10.3389/fonc.2021.627217

**Published:** 2021-04-07

**Authors:** Judith Penkert, Andre Märtens, Martin Seifert, Bernd Auber, Katja Derlin, Ursula Hille-Betz, Philipp Hörmann, Norman Klopp, Jana Prokein, Lisa Schlicker, Frank Wacker, Hannah Wallaschek, Brigitte Schlegelberger, Karsten Hiller, Tim Ripperger, Thomas Illig

**Affiliations:** ^1^Department of Human Genetics, Hannover Medical School, Hannover, Germany; ^2^Department of Bioinformatics and Biochemistry, Braunschweig Integrated Center of Systems Biology (BRICS), Technische Universität Braunschweig, Braunschweig, Germany; ^3^Connexome Consulting, Fischen, Germany; ^4^Department of Diagnostic and Interventional Radiology, Hannover Medical School, Hannover, Germany; ^5^Department of Obstetrics and Gynecology, Hannover Medical School, Hannover, Germany; ^6^Hannover Unified Biobank (HUB), Hannover, Germany; ^7^Center for Information Management, Hannover Medical School, Hannover, Germany; ^8^Division of Tumour Metabolism and Microenvironment, German Cancer Research Center (DKFZ), Heidelberg, Germany; ^9^Computational Biology of Infection Research, Helmholtz Centre for Infection Research, Braunschweig, Germany

**Keywords:** breast cancer, plasma metabolome, *BRCA1* germline mutation, energy metabolism, NAD^+^ balance, HIF1 alpha, lactate, aerobic glycolysis

## Abstract

Individuals carrying a pathogenic germline variant in the breast cancer predisposition gene *BRCA1* (g*BRCA1*+) are prone to developing breast cancer. Apart from its well-known role in DNA repair, BRCA1 has been shown to powerfully impact cellular metabolism. While, in general, metabolic reprogramming was named a hallmark of cancer, disrupted metabolism has also been suggested to drive cancer cell evolution and malignant transformation by critically altering microenvironmental tissue integrity. Systemic metabolic effects induced by germline variants in cancer predisposition genes have been demonstrated before. Whether or not systemic metabolic alterations exist in g*BRCA1*+ individuals independent of cancer incidence has not been investigated yet. We therefore profiled the plasma metabolome of 72 g*BRCA1*+ women and 72 age-matched female controls, none of whom (carriers and non-carriers) had a prior cancer diagnosis and all of whom were cancer-free during the follow-up period. We detected one single metabolite, pyruvate, and two metabolite ratios involving pyruvate, lactate, and a metabolite of yet unknown structure, significantly altered between the two cohorts. A machine learning signature of metabolite ratios was able to correctly distinguish between g*BRCA1*+ and controls in ~82%. The results of this study point to innate systemic metabolic differences in g*BRCA1*+ women independent of cancer incidence and raise the question as to whether or not constitutional alterations in energy metabolism may be involved in the etiology of *BRCA1*-associated breast cancer.

## Introduction

During recent years, the idea of the tumor suppressor and breast cancer predisposition gene *BRCA1* and its protein product exclusively serving as a critical DNA repair agent maintaining genomic stability has been challenged, as it has become increasingly apparent that BRCA1’s pleiotropic functions comprise mechanisms as widespread as epigenetic regulation (1), chromatin remodeling and gene transcription (2, 3), differentiation of mammary stem/progenitor cells to mature luminal epithelial cells (4), control of cancer stem cell-like characteristics (5), and the powerful regulation of cellular metabolism (6–13).

At the same time, the emerging concept of cancer initiation and progression being an evolutionary process in which not only the accumulation of mutational burden in tumor cells but also tissue integrity and alterations in stem cell niche microenvironments play a fundamental role (14) gains stronger interest. In this context, progressively degrading tissues, e.g. due to aging processes, chronic pro-inflammatory status, or external insults such as radiation, represent a challenge for resident stem cells poorly adapted to such niche changes and provide a competitive advantage to those that improve their fitness through specific oncogenic mutations (a process termed “adaptive oncogenesis”). For the emergence of altered microenvironment conditions, metabolic cellular reprogramming of stromal cells such as cancer-associated fibroblasts (CAFs) plays an integral role [reviewed in (15)]. Therefore, it seems conceivable that germline alterations that constitutionally push the organism towards a metabolic state resembling aging conditions or chronic inflammation amount to a microenvironment that—in advance of natural aging—supports adaptive oncogenesis and, thus, contributes to cancer predisposition.

As has previously been shown for recognized “Inborn Errors of Metabolism”, metabolic alterations such as succinate dehydrogenase (SDH) or fumarate hydratase (FH) deficiency are capable of fostering malignant transformation efficiently and reliably, and heterozygous germline alterations in the respective genes are associated with cancer predisposition syndromes [reviewed in (16)]. Not only has metabolic cellular reprogramming been named a hallmark of cancer (17), but emerging evidence seems to also raise the question if disrupted metabolism may in fact be a prerequisite for cancer evolution. BRCA1, specifically, has been shown to strongly impact energy metabolism, fatty acid metabolism, and antioxidative pathways in breast epithelial cells and breast cancer cell lines (11–13). A reversion of the Warburg effect has been postulated upon transfection of a *BRCA1*-mutated breast cancer cell line by wildtype *BRCA1* (11). In CAFs, BRCA1 has been shown to influence proliferation rates, response to hypoxia, autophagy, and SDH complex efficiency (18). Many of these effects seem to involve the transcription factor and master regulator of metabolism hypoxia-inducible factor 1 (HIF1) (11, 12, 18), the overexpression of which has been demonstrated in *BRCA1*-related invasive breast cancer as well as ductal carcinoma *in situ* (DCIS), suggesting hypoxia to already play a role in early stages of *BRCA1*-related breast carcinogenesis (19, 20).

While little is known about systemic effects of heterozygous pathogenic germline *BRCA1* variants (g*BRCA1*+), luteal phase sex hormones—in particular progesterone (P4)—were observed to be elevated and osteoprotegerin (OPG) levels were decreased in serum of g*BRCA1*+ carriers (21, 22). In women without known genetic predisposition, high receptor activator of NFκB ligand (RANKL)/OPG ratios were suggested indicative of breast cancer manifestation, and elevated RANKL and P4 serum levels stratified a subgroup of women at high risk of developing breast cancer 1–2 years before diagnosis (23), though recently a report rebutted plasma RANKL levels correlating with breast cancer risk in germline *BRCA1/2* mutation carriers (24). In patients with triple-negative breast cancer (TNBC), a plasma metabolomics signature has been described, which distinguishes diseased g*BRCA1*+ patients from diseased germline *BRCA1* wildtype patients (25). Whether or not systemic effects of globally altered metabolism exist in non-cancer-diseased g*BRCA1*+ individuals and whether these can be detected *via* metabolome analyses has, to our knowledge, not yet been investigated.

The purpose of the present study was to evaluate if plasma metabolome signatures of non-cancer-diseased g*BRCA1*+ carriers and healthy age-matched controls mirror genotype, as has previously been shown for carriers of pathogenic germline *PTEN* variants (26). We intentionally selected for non-diseased, treatment-naïve females seeking to confine confounding factors such as tumor burden, radio-chemotherapy, or anti-hormonal treatment to a minimum to allow primary focus on genotype-related metabolic differences. Findings of distinct systemic metabolic alterations preceding tumor formation could not only have vast consequences in terms of diagnostic procedures, predictive measures, and prognostic assessment, but also could they provide clues to etiologically critical drivers of cancer evolution in g*BRCA1*+ carriers and potentially uncover preventive as well as therapeutic options.

## Materials and Methods

### Study Cohort

#### Patient Selection

Plasma samples of 72 women carrying a heterozygous (likely) pathogenic germline variant in *BRCA1* (ACMG criteria class 4/5)—but not previously diagnosed with any type of malignancy—were recruited from the Departments of Human Genetics, Radiology, or Obstetrics and Gynecology, Hannover Medical School, during 2015 to 2018. All of the study subjects’ pedigrees of at least three generations had been established during genetic counseling. Subjects who knowingly carried an additional (likely) pathogenic variant (PV) in any other established cancer predisposition gene were dismissed from study inclusion.

In order to diminish the possibility of metabolomics effects arising from potentially pre-existing breast cancer disease in g*BRCA1*+ individuals at the time of blood draw, patients who developed invasive or *in situ* breast cancer during the follow-up period were excluded from the cohort. The follow-up period comprised a minimum of 19 months and a maximum of 56 months. For 14 g*BRCA1*+ women (19%), no clinical follow-up data was available.

Female relatives of patients in whom a PV in an established predisposition gene associated with Mendelian disease had previously been detected and who tested negative for this familial variant were included as age-matched control subjects (i.e., +/- 5 years, in one pair 6 years, at time of sample collection; 42/72 (~60%) of pairs being age-matched ≤2 years). As these individuals had also been seen in the outpatient clinic of the Department of Human Genetics, their personal medical and family histories were available, which enabled us to specifically exclude any sporadic cancer patients or any whose family history was suggestive of cancer predisposition in the other family branch according to currently available genetic testing criteria. Known carriers of variants of uncertain significance (ACMG class 3) or PVs in other genes were excluded. Among the included control subjects, there were 33, 24, 3, 2, and two in which a familial PV in *BRCA1*, *BRCA2*, *MSH2*, *MSH6*, and *TP53*, was excluded, respectively, and eight with exclusion of a familial PV in either *APC*, *CFTR, FBN1*, *GLI3*, *MLH1*, *PALB2*, *PTEN*, or of a duplication of 22q11. No clinical follow-up data were available for control subjects.

#### Cohort Characteristics

**Figure 1:**
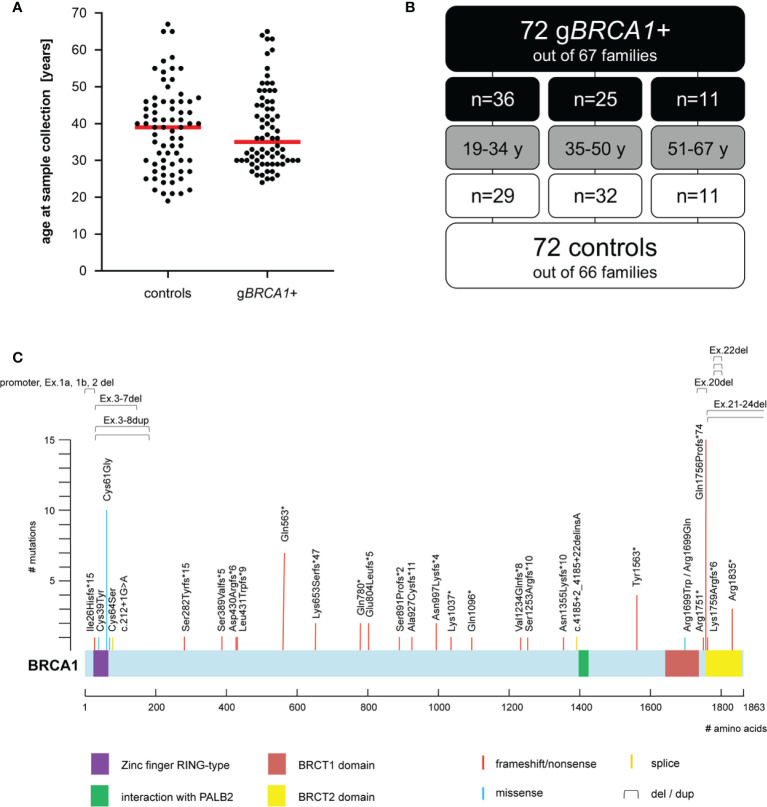
Cohort characteristics. **(A)** Age distribution at time of plasma sampling; median depicted as horizontal red line. **(B)** Age composition of cohort. **(C)** Overview of (likely) pathogenic *BRCA1* variants within the cohort (i.e., five exonic deletions, one exonic duplication, 15 frameshift, seven nonsense, five missense, and two splice-site variants). *BRCA1* NCBI reference sequence NM_007294.2; UniProtKB - P38398 (BRCA1_HUMAN).

The final cohort (**Figures 1A, B****)** consisted of 144 individuals from 130 different families, including three families out of which cases and controls were integrated. The age range of cases and controls was 24–65 years (median 35 years), and 19–67 years (median 39 years), respectively. For age-stratified analyses, the cohort was split into three distinct age groups: G1: 19–34 years, G2: 35–50 years, and G3: 51–67 years. The g*BRCA1*+ cohort contained 35 distinct variants in the *BRCA1* gene (for details, see **Figure 1C**).

The majority of patients and controls were assumed to be of European-Caucasian ancestry. The present study was performed in accordance with the Declaration of Helsinki and was approved by the institutional ethics review board of Hannover Medical School. Written informed consent was obtained from all participants.

### Sample Processing and Gas Chromatography-Mass Spectrometry Measurement

#### Sample Collection

Peripheral blood EDTA samples were transferred to Hannover Unified Biobank (HUB) in a cool box immediately after blood draw. For plasma separation, samples were centrifuged at 2.000 x g for 10 min at 4°C and aliquoted within 2 h from blood collection. Plasma aliquots were immediately stored at −192°C. Samples were stored and tracked according to standard operating procedures at HUB and sent to Technical University, Braunschweig, on dry ice for further analyses.

#### Metabolite Extraction

All blood plasma samples were processed in technical duplicates. For each replicate, 20 μl of plasma were mixed with 180 μl methanol/water mixture (4 + 1; v/v) and vortexed on a thermomixer (Eppendorf) for 5 min at 1.400 rpm at 4°C. The mix was then directly centrifuged at 17.000 x g for 5 min at 4°C (Eppendorf 5415R), and 140 μl of the supernatant were transferred into GC glass vials. Samples were completely dried in a refrigerated rotary vacuum evaporator (Labconco) at 4°C for at least 40 min. In order to avoid condensation of water on the glass surface of the vials, the refrigerated rotary vacuum evaporator was heated up to room temperature for 25 min prior to taking out the vials. In addition, plasma metabolite pools (quality controls) were produced during the metabolite extraction procedure by mixing an equal amount of each sample. The GC glass vials were stored at −80°C until GC-MS measurements.

#### Metabolite Derivatization and GC-MS Analysis

Derivatization of the samples was performed by an autosampler (Axel Semrau) directly before GC-MS measurement. The dried samples were dissolved in 15 μl methoxyamine hydrochloride (Sigma-Aldrich) in pyridine (20 mg/ml) at 40°C for 60 min under shaking, followed by addition of 15 μl N-Methyl-N-(trimethylsilyl)trifluoroacetamide (Macherey-Nagel) and subsequent incubation for 30 min at 40°C.

GC-MS measurements were performed on a 7890B GC coupled to a 5977B MSD (both Agilent Technologies). The gas chromatograph was equipped with a 30 m DB-35ms capillary column (I.D. 250 µm, film 0.25 µm) + 5 m DuraGuard capillary in front of the analytical column (Agilent J&W GC Column). A sample volume of 1 µl was injected into a split/splitless inlet, operating in splitless mode at 270°C. Helium was used as carrier gas with a constant flow rate of 1 ml/min. The GC oven temperature was held at 80°C for 6 min, ramped with 6°C/min to 300°C and was held for 10 min. Then, the temperature was increased to 325°C at 10°C/min and held for additional 4 min. The total run time was 59.167 min. The transfer line temperature was set to 280°C. The MSD was operating under electron ionization at 70 eV. The MS source was held at 230°C and the quadrupole at 150°C. Full scan mass spectra were acquired from m/z 70 to m/z 800. Pool samples have been measured after 8 GC-MS measurements for quality control and data correction.

### Data Processing, Statistical Analysis, and Machine Learning

All GC-MS chromatograms were processed using MetaboliteDetector, v3.320200313 (27). The software package supports automatic deconvolution of all mass spectra. Compounds were annotated by retention time and mass spectrum using an in-house mass spectral library. Data normalization was performed by dividing each metabolite intensity by the median intensity of the three chronologically nearest pool samples of the corresponding metabolite. After pool normalization, a total of 262 metabolites have been selected for further analysis after filtering of GC-MS measurement artefacts, such as siloxanes.

Normalized data were subject to statistical analysis using Python (version 3.7.6). To diminish potential deteriorating effects of outliers, mal-assigned signals, and artefacts on statistical analysis, we excluded all metabolites featuring a relative standard deviation (RSD) of ≥20%. We performed a two-tailed Welch’s t-test (scipy version 1.4.1) as well as correction for multiple testing by Benjamini & Hochberg p-value adjustment (statsmodels version 0.11.0), the designed level of significance being p<.05. Metabolite ratios were determined per patient and replicate, means for both replicate-ratios were generated for each patient, and subsequently the two cohorts were compared.

For machine learning approaches we used the Waikato Environment for Knowledge Analysis (Weka) (https://www.cs.waikato.ac.nz/ml/weka/). Weka is a workbench for machine learning that is intended to aid in the application of machine learning techniques to a variety of data mining problems in bioinformatics research (28). Different machine learning algorithms including random forest, J48 simple logistic, and SMO (Sequential Minimal Optimization) – a training algorithm of support vector machines – were evaluated. Each classification method was used with Weka’s default settings. We mainly performed Simple Logistic Regression analyses, which are frequently applied for cancer classification issues. Simple Logistic in Weka fits a multinomial logistic regression model using the LogitBoost algorithm (29). The number of LogitBoost iterations was manually selected based on an optimization of cross validation results. To build a classifier *via* stratified cross validation (training), we used 1) raw data, 2) averaged data (mean from both replicates per metabolite and patient), 3) averaged data of metabolites with RSD <20%, 4) metabolite ratios (see above), and 5) metabolite ratios plus metabolites with RSD <20% for different sub-analyses. The resulting classifiers were subsequently applied to a) the whole dataset, and b) the separate age-subgroups (re-evaluation on test set).

## Results

### Differentially Expressed Metabolites Between g*BRCA1*+ Carriers and Controls

**Figure 2:**
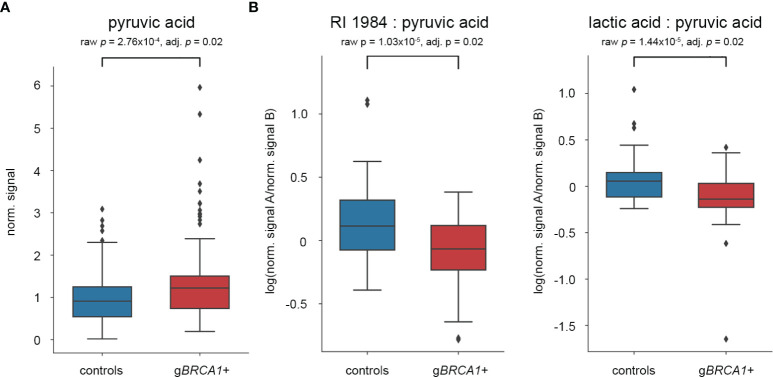
Results of statistical analyses after multiple testing correction. Box plots of **(A)** pyruvic acid as single plasma metabolite, and **(B)** two metabolite ratios significantly differing between g*BRCA1*+ individuals and controls.

We profiled the plasma metabolome of 72 g*BRCA1*+ and 72 healthy controls in technical duplicates. The analysis yielded 262 metabolites with unique retention time and mass spectrum detectable across all subjects (**Supplementary Material 1**). After excluding all metabolites featuring an RSD of ≥20%, 78 out of 262 identifiable metabolites remained within the analysis (**Supplementary Material 2**). From these, we identified three metabolites with significantly differing concentrations between g*BRCA1*+ and control subjects. After applying multiple testing correction *via* Benjamini-Hochberg adjustment, pyruvic acid was left as the sole significantly different metabolite, elevated in g*BRCA1*+ (**Figure 2A**).

### Differentially Expressed Metabolite Ratios Between g*BRCA1*+ Carriers and Controls

Alterations of pairwise metabolite ratios are often more informative about specific metabolic pathways and disease mechanisms. Including all 78 metabolites with an RSD <20%, the resulting matrix consisted of 3.003 possible pairwise ratios. Of these, 208 metabolite ratios significantly differed between g*BRCA1*+ and control subjects. After Benjamini-Hochberg adjustment, two metabolite ratios remained significant, specifically RI1984:pyruvic acid and lactic acid:pyruvic acid, both reduced in g*BRCA1*+ carriers (**Figure 2B**). Although we have not been able to elucidate the structure of RI1984, its mass spectrum suggests a sugar acid. Based on a reference measurement, we excluded gluconic acid as candidate metabolite.

### Machine-Learning Signature

**Figure 3:**
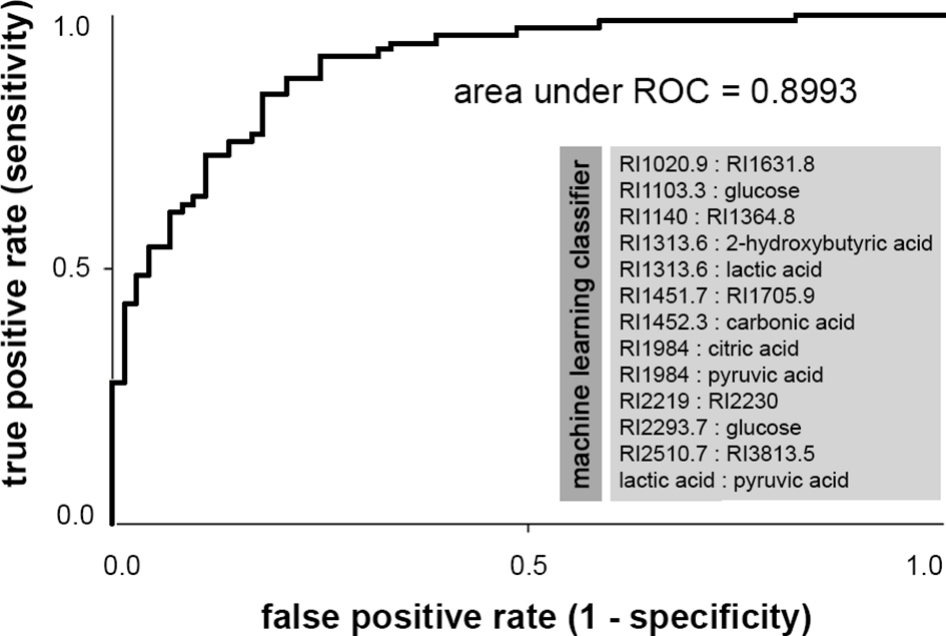
Receiver operating characteristic (ROC) curve of metabolite ratios classifier for the prediction of *BRCA1* genotype, obtained by machine learning. Area under ROC is a performance metric used to evaluate classification models that classify subjects into one of two categories. The ROC curve is created by plotting the true positive rate (i.e., sensitivity) against the false positive rate (i.e., 1-specificity). Smaller values on the x-axis of the plot indicate lower false positives and higher true negatives. Larger values on the y-axis of the plot indicate higher true positives and lower false negatives. Area under ROC equals 0.5 when the ROC curve corresponds to random chance and the model allows no classification. An area under ROC of 1.0 means perfect accuracy (i.e., sensitivity and specificity being 100%). Generally, an area under ROC greater than 0.8 indicates very good classification performance. All metabolite ratios included in the machine learning classifier are listed in the light gray box below the curve. This classifier correctly classifies ~82% of the cohort per genotype (specificity: 0.79, sensitivity: 0.85).

Additionally, we applied a machine learning approach in order to disclose complex interrelations between metabolites and metabolite ratios for genotype prediction. A 10-fold cross validation strategy was applied. Best results were obtained for the classifier based on pairwise metabolite ratios. After training of the Simple Logistic Model with this data set, we obtained 63.9% correct classification of samples per genotype. Using this classifier to evaluate the entire dataset, 81.9% of subjects (specificity: 0.79, sensitivity: 0.85) were assigned the correct genotype (**Figure 3**). Evaluation of the three age-subgroups separately yielded 80.0%, 84.2%, and 81.8% correct classification of G1, G2, and G3, respectively.

The classifier resulting from training on ratios plus averaged data of metabolites with RSD <20% exclusively contained metabolite ratios, indicating that metabolite ratios are more informative and perform better than single metabolite data. All classifiers generated contained the elements pyruvic acid, lactic acid, and RI1984, except for the classifier based on averaged data, which did not include lactic acid. In all sub-analyses, the classifiers consistently performed best on the middle age group G2, which included subjects aged 35–50 years at time of plasma sampling. Given the 10-fold cross validation strategy, we tried to avoid over-fitting. For an overview of all classifiers produced by sub-analyses, see **Supplementary Material 3**. For complete raw data, see **Supplementary Material 4**.

## Discussion

Inquiring an organism’s plasma metabolome represents a unique challenge distinct from investigating primary tissue of interest. As such, the plasma metabolome can be seen as a dynamic equilibrium between total tissue consumption of specific metabolites, total flux from tissue into bloodstream (including endogenous production rates of metabolites), intestinal absorption of metabolites, and excretion *via* elimination mechanisms such as bile acid secretion or kidney filtration.

In comparison with age-matched controls, we detected two metabolite ratios significantly reduced in plasma of g*BRCA1*+ carriers after multiple testing correction, i.e., lactic acid:pyruvic acid and RI1984:pyruvic acid. Of the involved metabolites, pyruvate showed the most significant deviation as a single metabolite. Although the differences were not highly statistically significant, we believe that even discreet metabolic plasma effects justify appropriate notice in a study cohort fully consisting of healthy individuals lacking a personal history of cancer. As effect sizes were not particularly strong, we additionally applied a machine learning approach as a second method, which resulted in the correct discrimination between g*BRCA1*+ and controls in ~82% *via* a metabolite ratios signature that included both ratios previously identified as significantly different. Since no breast cancer was observed in g*BRCA1*+ subjects within a minimum follow-up period of 19 months, it seems unlikely that metabolic effects depicted in this cohort are attributable to preclinical undiagnosed breast cancer incidence in g*BRCA1*+ women. Because clinical follow-up data were not available for control subjects, breast cancer incidence during follow-up of g*BRCA1*+ cases cannot be ruled out for the control cohort. However, as we had excluded all subjects whose family pedigree suggested another cancer predisposition or who had previously been diseased, our control cohort was extremely carefully composed and potentially co-occurring malignancies should constitute rare events. The results of this study therefore point to innate systemic metabolic differences in g*BRCA1*+ individuals independent of and preceding cancer incidence, raising the disputable question as to whether or not metabolic alterations may pave the way for cancer evolution in g*BRCA1*+ individuals.

**Figure 4:**
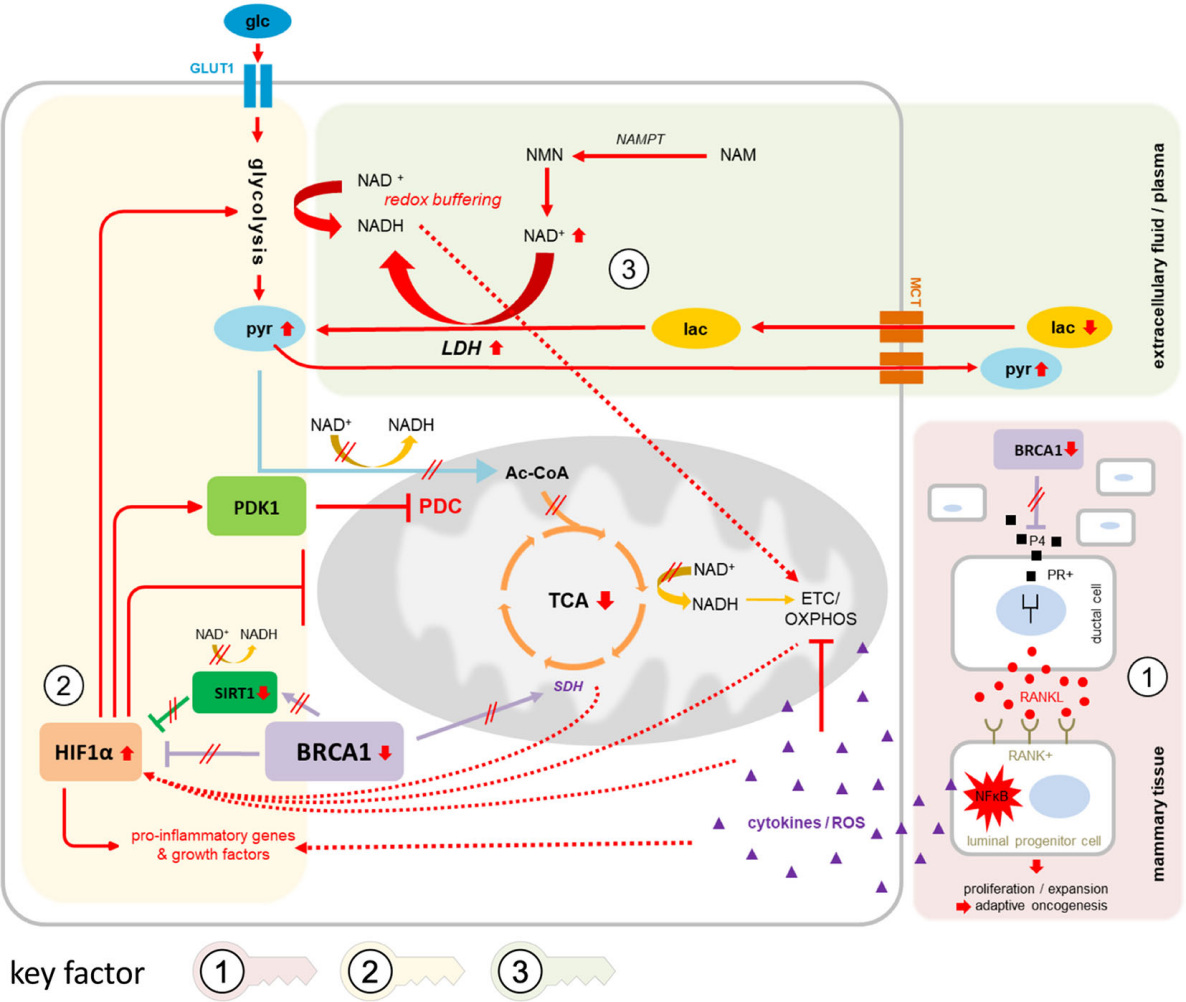
Suggested network of contributing factors in g*BRCA1*+ oncogenesis. Circled numbers/shaded areas = key factors. In g*BRCA1*+ precancerous mammary tissue (key factor 1, red shading), amplification of the progesterone signaling axis due to elevated P4 levels and heightened PR activity in BRCA1-low conditions mediate RANKL secretion in PR+ ductal cells and subsequent paracrine binding to corresponding RANK on RANK+ luminal progenitor cells (LPC). In these cells, NFkB signaling is strongly activated, leading—in parallel—to i) chronic pro-inflammatory, ROS-enriched microenvironmental conditions, which contribute to OXPHOS impairment and mitochondrial dysfunction, eventually resulting in declined tissue integrity supportive of cancer evolution, and ii) a strong pro-proliferative signal specifically in RANK+ LPCs, increasing the chances of eruption of malignant phenotypes that are properly adjusted to challenging environmental conditions (the process of adaptive oncogenesis). Hypoxic as well as pseudohypoxic triggers mediate the stabilization and activation of HIF1A (key factor 2, yellow shading), amplified through direct and indirect interactions *via* BRCA1 as well as BRCA1-mediated alterations in SIRT1 and SDH activities. Downstream effects of HIF1A activation cause i) a massive increase in glycolytic flux *via* transcriptional activation of glycolytic enzymes and GLUT1, ii) further reinforcement of mitochondrial dysfunction and impaired OXPHOS, e.g. *via* TFAM iii) induction of pro-inflammatory genes and growth factors, and iv) strong activation of PDK1 resulting in impaired conversion of pyruvate to acetyl-CoA and, thus, diminished fuel for TCA cycling and energy generation from three-carbon compounds. The equilibrium between pyruvate and lactate *via* LDH, i.e., the direction of net LDH flux, is governed by cellular NAD^+^/NADH ratios (key factor 3, green shading); as NAD^+^ levels are elevated in BRCA1-low conditions due to upregulated NAMPT-mediated synthesis, lactate may be more readily converted into pyruvate than vice versa. Lactate influx and pyruvate efflux through MCTs would therefore mediate redox buffering of intracellular NAD^+^/NADH ratios while affecting net pyruvate and lactate levels in systemic circulation. NADH generated from glycolysis and from conversion of lactate into pyruvate may immediately be transported into mitochondria *via* malate-aspartate or glycerol phosphate shuttles to feed the otherwise neglected ETC for energy generation. 3PG, 3-phosphoglycerate; Ac-CoA, acetyl coenzyme A; αKG, alpha-ketoglutarate; ETC, electron transport chain; F1,6BP, fructose 1,6-bisphosphate; F6P, fructose 6-phosphate; fum, fumarate; G6P, glucose 6-phosphate; glc, glucose; GLUT1, glucose transporter 1; HIF1A, hypoxia-inducible factor 1 alpha; HK2, hexokinase 2; IL-6, interleukin 6; lac, lactate; LDH, lactate dehydrogenase; MCT, monocarboxylate transporter; NAD^+^, nicotinamide adenine dinucleotide (oxidized form); NADH, nicotinamide adenine dinucleotide (reduced form); NAM, nicotinamid; NFκB, nuclear factor “kappa-light-chain-enhancer” of activated B-cells; NMN, nicotinamide mononucleotide; OAA, oxaloacetate; OXPHOS, oxidative phosphorylation; P4, progesterone; PDC, pyruvate dehydrogenase complex; PDK1, pyruvate dehydrogenase kinase 1; PEP, phosphoenolpyruvate; PFK2, phosphofructokinase 2; PKM2, pyruvate kinase isozyme M2; PR+, progesterone receptor positive; pyr, pyruvate; RANK+, Receptor Activator of NFκB positive; RANKL, Receptor Activator of NFκB ligand; ROS, reactive oxygen species; SDH, succinate dehydrogenase; SIRT1, sirtuin 1; succ, succinate; TCA, tricarboxylic acid cycle; TFAM, mitochondrial transcription factor A; TNFα, tumor necrosis factor alpha; VEGF, vascular endothelial growth factor.

The observed metabolic changes can be interpreted within the scope of a network of alterations already known to correlate with BRCA1-low conditions (**Figure 4**): NFkB signaling is induced in precancerous breast tissue of g*BRCA1*+ carriers *via* paracrine signaling mechanisms involving P4-RANKL (30–32) (**Figure 4**, key factor 1 ), leading to pro-inflammatory stromal conditions that augment hypoxia-inducible factor 1 alpha (HIF1A) transcription (33)—one of two subunits constituting the transcription factor HIF1. Upregulation of HIF1A in BRCA1-deficient fibroblasts has previously been shown to drive breast cancer growth (18). In addition to upregulated transcription, HIF1A is stabilized in BRCA1-low conditions *via* impairment of two of four subunits of the mitochondrial SDH complex and subsequent accumulation of the oncometabolite succinate (11, 18), and *via* suppressed sirtuin 1 (SIRT1) levels (34) mimicking a pseudohypoxic state that results in loss of mitochondrial homeostasis (35). HIF1 (**Figure 4**, key factor 2 ) acts as a master transcriptional regulator of metabolism mediating i) massively increased glycolytic flux *via* transcriptional activation of glycolytic enzymes and glucose transporter 1 (GLUT1), ii) a reduction in mitochondrial-encoded gene expression, resulting in reduced mitochondrial function and impaired OXPHOS (35), iii) activation of pro-inflammatory genes and growth factors such as TNFα, interleukin 6 (IL-6), and VEGF (36), and iv) strong activation of pyruvate dehydrogenase kinase 1 (PDK1) (37), which regulates pyruvate dehydrogenase complex (PDC), resulting in reduced conversion of pyruvate to acetyl-CoA and, thus, pyruvate accumulation. The lack of glycolytic acetyl-CoA as the major substrate for the TCA cycle results in its decrement and further suppression of mitochondrial respiration. In summary, HIF1 regulates multiple genes contributing to the “Warburg effect”—a metabolic switch towards glycolytic over oxidative metabolism under normoxic conditions, i.e., “aerobic glycolysis” [reviewed in (38)].

Moreover, NAD^+^ levels are known to be elevated in BRCA1-low conditions, partly due to upregulated nicotinamide phosphoribosyltransferase (NAMPT)-mediated NAD^+^ synthesis (**Figure 4**, key factor 3 ), and increased NAD^+^ levels as well as elevated NAD^+^/NADH ratios have been shown to activate *BRCA1* transcription in turn (39, 40). The equilibrium between pyruvate and lactate is governed by cellular NAD^+^/NADH ratios, as are multiple other reversible enzymatic reactions, and it has been suggested that redox buffering of intracellular NAD^+^/NADH ratios across cells and tissues could take place *via* uptake regulation of lactate or pyruvate through nearly universally expressed MCT transporters from systemic circulation (41). Thus, in such a setting of excessive NAD^+^ over NADH, cellular net uptake of lactate and simultaneous excretion of pyruvate would help alleviate intracellular redox imbalance. Notably, this scenario would require high lactate dehydrogenase (LDH) activity, the expression of which was indeed shown to be upregulated in a BRCA1-mutated cell line (11). The generated NADH, in turn—both scarce and valuable due to low NADH output through TCA cycling—would likely be instantly shuttled into mitochondria *via* malate-aspartate or glycerol-3-phosphate shuttles in an attempt to produce energy *via* OXPHOS, further perpetuating increased cellular NAD^+^/NADH ratios. The significantly elevated pyruvate levels and reduced lactate:pyruvate ratios in plasma of g*BRCA1*+ individuals observed in our study may reflect on these alterations in co-factor balance and equilibrium adjustment.

In line with previously published data on *BRCA1*-driven metabolic rewiring, our results support a bioenergetic shift in g*BRCA1*+ individuals towards aerobic glycolysis, traceable even in the plasma metabolome of healthy g*BRCA1*+ carriers. Within the context of “adaptive oncogenesis,” in which stromal tissue degradation sets the stage for cancer cell evolution *via* selection for adaptive phenotypes in heterogeneous populations of stem or progenitor cells (14), an excessively anabolic, pro-inflammatory, and pro-proliferative environment producing massive amounts of macromolecules from glycolysis and shuttling them to surrounding cells could provide the necessary growth advantage to adjacent (pre)-malignant cells that acquired the ability to import and thrive on these nutrients [a phenomenon known as the “reverse Warburg effect,” reviewed in (15)].

The strengths of this study include a well-characterized cohort of g*BRCA1*+ and age-matched control subjects, comprising established family pedigrees as well as individual clinical information. We have collected standardized and documented high-quality biosamples, which were frozen within 2 h of blood draw. We were able to carefully select control subjects pursuant to their bland familial backgrounds, which contrasts conventional practice and is a privilege of this particular study. To avoid confounding factors, none of the study subjects had previously been diagnosed with malignant disease, nor were any diagnosed during the follow-up period.

Limitations include the following:

- Only two technical replicates per sample and no biological replicates were analyzed.
- Due to relatively small study size, the method is susceptible to errors, overfitting can occur, and study power is limited.
- Many metabolites lack a clear identity.
- Samples were not collected during identical menstrual cycle phases, e.g. luteal phase in which P4 levels are physiologically elevated and effects might be stronger.
- No clinical follow-up data were available for control subjects.
- Results were not validated in an independent validation cohort.

Future validation in an independent cohort and proteomics/transcriptomics analyses on plasma as well as metabolomics and other -omics analyses on breast tissue of g*BRCA1*+ carriers (preferably single cell analyses) are instrumental in further addressing currently hypothetic pathogenic mechanisms. Importantly, if proven correct, these complex disease mechanisms would yield multiple options for therapeutic targets and preventative measures in g*BRCA1*+ carriers. Moreover, the classifier prediction model of *BRCA1*+ status, if validated and enhanced, could have great implications for diagnostic genotype prediction independent of genetic testing or complementation of genetic testing in case of non-conclusive results, for personalized risk assessment, and for individual clinical measures.

## Data Availability Statement

The datasets presented in this study can be found in online repositories. The names of the repository/repositories and accession number(s) can be found in the article/**Supplementary Material**.

## Ethics Statement

The studies involving human participants were reviewed and approved by the institutional ethics review board of Hannover Medical School. The patients/participants provided their written informed consent to participate in this study.

## Author Contributions

JPe, TR, and TI contributed to conception and design of the study. BS, BA, HW, KD, FW, and UH-B were involved in resource acquisition. NK and JPr organized the database. LS, PH, AM, and KH contributed to the acquisition of data. AM, MS, and KH performed the statistical analysis. JPe wrote the original draft of the manuscript. All authors contributed to the article and approved the submitted version.

## Funding

TR was supported by the intramurally funded Clinical Scientist program of Hannover Medical School.

## Conflict of Interest

MS was employed by the company Connexome Consulting.

The remaining authors declare that the research was conducted in the absence of any commercial or financial relationships that could be construed as a potential conflict of interest.
