# Supplementary material for: Plasma Metabolome Signature Indicative of BRCA1 Germline Status Independent of Cancer Incidence
Source: Front Oncol. 2021 Apr 7;11:627217. doi: 10.3389/fonc.2021.627217 (PMC8058469; doi:10.3389/fonc.2021.627217)
Supplement: Supplementary file 1 [file Table_1.docx]

**Supplementary Material 1.** List of mean ± standard deviation of all 262 identifiable metabolites; p-values were calculated using a two-tailed Welch’s t-test. For metabolites identified via mass spectrometry metabolite libraries, metabolite names and derivatization details (MeOX: methoxyamine, TMS: trimethylsylyl) are specified. If metabolites had more than one peak, the main product is denoted as ‘MP’ and byproducts as ‘BP’. For complete raw data, see Supplementary Material 4.

| **metabolite** | **controls** | **g*BRCA1*+** | **p-value** |
| --- | --- | --- | --- |
| 1,5-anhydro-D-glucitol 4TMS | 0.92 ± 0.83 | 0.97 ± 0.49 | 0.5143 |
| 2-hydroxybutyric acid 2TMS | 1.26 ± 0.97 | 1.06 ± 0.76 | 0.0634 |
| 3-hydroxybutyric acid 2TMS | 1.41 ± 1.90 | 1.18 ± 1.45 | 0.2517 |
| adenine 1TMS | 1.11 ± 0.75 | 0.93 ± 0.65 | 0.0293 |
| arginine 3TMS | 1.10 ± 0.83 | 1.21 ± 1.15 | 0.3588 |
| aspartic acid 3TMS | 1.20 ± 0.54 | 1.17 ± 0.51 | 0.7385 |
| carbonic acid 1MeOX 2TMS | 0.97 ± 0.30 | 0.93 ± 0.27 | 0.2197 |
| cholesterol 1TMS | 0.84 ± 0.18 | 0.83 ± 0.17 | 0.6413 |
| citric acid 4TMS | 0.98 ± 0.28 | 1.03 ± 0.30 | 0.1754 |
| dehydroascorbic acid dimer 2MeOX MP | 1.65 ± 2.52 | 1.38 ± 1.93 | 0.3093 |
| galactose 1MEOX 5TMS MP | 3.00 ± 4.54 | 3.24 ± 5.17 | 0.6708 |
| glucose 3TMS BP1 | 4.74 ± 5.71 | 4.62 ± 5.85 | 0.8608 |
| glucose 3TMS BP2 | 0.97 ± 0.78 | 1.08 ± 0.74 | 0.1975 |
| glucose 1MEOX 5TMS MP | 1.08 ± 0.19 | 1.07 ± 0.22 | 0.4865 |
| glucose 4TMS MP | 1.94 ± 3.63 | 1.30 ± 2.38 | 0.0816 |
| glucose 5TMS BP1 | 1.24 ± 1.36 | 1.16 ± 1.51 | 0.6604 |
| glucose 5TMS MP | 0.68 ± 0.35 | 0.69 ± 0.29 | 0.7549 |
| glutamic acid 3TMS | 0.95 ± 0.73 | 0.99 ± 0.59 | 0.6633 |
| glutamine 3TMS | 1.27 ± 1.99 | 1.23 ± 1.93 | 0.8810 |
| glycerol 3TMS | 1.15 ± 0.41 | 1.24 ± 0.48 | 0.1001 |
| glycine 2TMS | 1.17 ± 0.53 | 1.18 ± 0.55 | 0.8634 |
| glycine 3TMS | 1.07 ± 0.38 | 1.15 ± 0.49 | 0.1030 |
| hexadecanoic acid 1TMS | 0.99 ± 0.44 | 0.96 ± 0.45 | 0.5438 |
| histidine 4TMS | 0.96 ± 0.59 | 1.02 ± 0.66 | 0.4333 |
| homocystine 4TMS | 1.07 ± 0.85 | 1.14 ± 0.90 | 0.5008 |
| isoleucine 2TMS | 1.03 ± 0.48 | 1.06 ± 0.50 | 0.5596 |
| lactic acid 2TMS | 1.01 ± 0.43 | 0.94 ± 0.33 | 0.1513 |
| lysine 4TMS | 1.05 ± 0.55 | 1.04 ± 0.48 | 0.9449 |
| mannose 5TMS BP | 0.79 ± 0.68 | 0.71 ± 0.54 | 0.2637 |
| mannose 5TMS MP | 1.07 ± 1.46 | 0.95 ± 1.01 | 0.4567 |
| methionine 2TMS | 0.85 ± 0.45 | 0.90 ± 0.48 | 0.3559 |
| myo-inositol 6TMS | 0.95 ± 0.49 | 0.97 ± 0.50 | 0.7585 |
| N-carboxy-L-lysine 4TMS | 0.74 ± 0.54 | 0.70 ± 0.42 | 0.4317 |
| N-carboxy-L-proline 2TMS | 1.13 ± 3.08 | 0.81 ± 0.80 | 0.2421 |
| N-carboxy-L-valine 3TMS | 1.15 ± 2.41 | 1.04 ± 0.99 | 0.5875 |
| N-carboxyasparagine 5TMS | 1.00 ± 0.77 | 1.10 ± 0.88 | 0.3032 |
| octadecanoic acid 1TMS | 1.01 ± 0.73 | 1.00 ± 0.75 | 0.8694 |
| ornithine 4TMS | 1.06 ± 0.53 | 1.08 ± 0.57 | 0.8144 |
| oxalic acid 2TMS | 1.28 ± 3.52 | 1.06 ± 1.23 | 0.4829 |
| phenylalanine 2TMS | 1.00 ± 0.31 | 1.05 ± 0.33 | 0.1789 |
| phosphoric acid 3TMS | 0.94 ± 0.28 | 0.97 ± 0.25 | 0.3399 |
| proline 2TMS | 0.99 ± 0.70 | 1.07 ± 0.64 | 0.3012 |
| pyroglutamic acid 2TMS | 1.08 ± 0.31 | 1.11 ± 0.39 | 0.5312 |
| pyruvic acid 1MEOX 1TMS | 1.00 ± 0.61 | 1.33 ± 0.89 | 0.0003 |
| scyllo-inositol 6TMS | 1.01 ± 1.23 | 0.94 ± 1.02 | 0.6193 |
| serine 3TMS | 1.03 ± 0.37 | 1.06 ± 0.36 | 0.4742 |
| succinic acid 2TMS | 4.34 ± 10.54 | 4.33 ± 11.74 | 0.9980 |
| threitol 4TMS | 0.81 ± 1.08 | 2.22 ± 12.72 | 0.1888 |
| threonic acid 4TMS | 2.49 ± 1.64 | 2.58 ± 1.91 | 0.6728 |
| threonine 3TMS | 1.03 ± 0.97 | 1.02 ± 0.60 | 0.8803 |
| tryptophan 3TMS | 0.88 ± 0.35 | 0.90 ± 0.33 | 0.5478 |
| tyrosine 2TMS | 1.16 ± 0.74 | 1.28 ± 1.23 | 0.2996 |
| tyrosine 3TMS | 0.82 ± 0.43 | 0.85 ± 0.46 | 0.6540 |
| urea 2TMS | 1.04 ± 0.75 | 1.10 ± 0.84 | 0.5170 |
| valine 2TMS | 1.00 ± 0.31 | 1.02 ± 0.31 | 0.4201 |
| xylose 1MEOX 4TMS MP | 1.55 ± 1.74 | 1.88 ± 2.30 | 0.1681 |
| RI1000 | 1.22 ± 2.00 | 1.38 ± 1.62 | 0.4735 |
| RI1001.2 | 1.16 ± 0.92 | 1.08 ± 0.59 | 0.3502 |
| RI1010.4 | 1.14 ± 0.55 | 1.10 ± 0.43 | 0.4770 |
| RI1014.2 | 4.93 ± 37.65 | 1.52 ± 2.53 | 0.2821 |
| RI1020.1 | 0.99 ± 0.22 | 1.00 ± 0.22 | 0.8630 |
| RI1020.9 | 1.02 ± 0.23 | 1.02 ± 0.25 | 0.8172 |
| RI1023 | 4.46 ± 11.65 | 2.55 ± 7.65 | 0.1025 |
| RI1030.2 | 1.11 ± 0.85 | 1.07 ± 0.43 | 0.6693 |
| RI1032.2 | 0.98 ± 0.72 | 1.01 ± 0.87 | 0.7491 |
| RI1036.3 | 0.98 ± 0.21 | 0.99 ± 0.19 | 0.8716 |
| RI1040.8 | 1.00 ± 0.40 | 0.94 ± 0.32 | 0.1937 |
| RI1103.3 | 1.05 ± 0.39 | 1.07 ± 0.28 | 0.6818 |
| RI1112.6 | 1.05 ± 0.57 | 1.01 ± 0.24 | 0.4414 |
| RI1120.3 | 0.76 ± 0.37 | 0.75 ± 0.34 | 0.8514 |
| RI1140 | 0.95 ± 0.39 | 0.94 ± 0.31 | 0.6988 |
| RI1154 | 1.45 ± 1.76 | 1.29 ± 1.68 | 0.4336 |
| RI1156.8 | 1.06 ± 0.35 | 1.06 ± 0.50 | 0.9153 |
| RI1169 | 10.35 ± 21.23 | 8.23 ± 16.63 | 0.3478 |
| RI1173 | 1.06 ± 0.62 | 0.97 ± 0.45 | 0.1593 |
| RI1185 | 1.12 ± 0.84 | 1.14 ± 0.90 | 0.8315 |
| RI1187.9 | 1.70 ± 2.37 | 1.68 ± 1.70 | 0.9120 |
| RI1226.9 | 1.06 ± 0.81 | 1.26 ± 1.39 | 0.1298 |
| RI1231.6 | 2.63 ± 4.88 | 3.64 ± 8.58 | 0.2211 |
| RI1234.2 | 1.20 ± 0.86 | 1.11 ± 0.73 | 0.3348 |
| RI1236.6 | 1.22 ± 0.84 | 1.30 ± 1.01 | 0.4369 |
| RI1247.9 | 1.55 ± 4.74 | 1.19 ± 1.06 | 0.3688 |
| RI1249.1 | 1.05 ± 0.42 | 1.06 ± 0.46 | 0.8642 |
| RI1254.1 | 1.32 ± 1.25 | 1.30 ± 1.07 | 0.8843 |
| RI1258.9 | 1.09 ± 0.59 | 1.11 ± 0.60 | 0.7060 |
| RI1262.1 | 1.36 ± 1.08 | 1.41 ± 0.96 | 0.6462 |
| RI1271.3 | 1.06 ± 0.67 | 1.14 ± 0.86 | 0.3542 |
| RI1281.2 | 5.75 ± 21.43 | 4.70 ± 14.50 | 0.6298 |
| RI1287.2 | 0.98 ± 0.47 | 0.93 ± 0.35 | 0.3069 |
| RI1290.8 | 0.98 ± 0.36 | 0.98 ± 0.41 | 0.9211 |
| RI1301.5 | 2.86 ± 20.73 | 1.62 ± 6.65 | 0.4948 |
| RI1310.7 | 1.12 ± 0.72 | 1.06 ± 0.52 | 0.4165 |
| RI1313.6 | 1.05 ± 0.61 | 1.17 ± 0.50 | 0.0664 |
| RI1313.9 | 5.91 ± 14.52 | 6.01 ± 13.58 | 0.9490 |
| RI1358.4 | 1.41 ± 1.38 | 1.38 ± 1.56 | 0.8689 |
| RI1360.4 | 1.13 ± 0.77 | 1.17 ± 0.89 | 0.6171 |
| RI1364.8 | 1.24 ± 1.19 | 1.12 ± 0.82 | 0.3393 |
| RI1367.8 | 4.18 ± 22.20 | 5.55 ± 37.09 | 0.7061 |
| RI1376.6 | 1.03 ± 0.25 | 0.98 ± 0.19 | 0.0587 |
| RI1378 | 5.53 ± 38.46 | 10.24 ± 70.08 | 0.4813 |
| RI1384.7 | 0.97 ± 0.38 | 0.93 ± 0.34 | 0.3544 |
| RI1384.8 | 1.10 ± 0.84 | 1.04 ± 0.66 | 0.4514 |
| RI1389.4 | 0.96 ± 0.22 | 1.00 ± 0.25 | 0.1328 |
| RI1393.1 | 1.23 ± 0.58 | 1.17 ± 0.61 | 0.3370 |
| RI1398.7 | 1.05 ± 0.48 | 1.11 ± 0.56 | 0.3235 |
| RI1405.9 | 1.37 ± 0.67 | 1.30 ± 0.55 | 0.3151 |
| RI1415.8 | 1.83 ± 1.80 | 1.49 ± 1.28 | 0.0632 |
| RI1439.6 | 1.44 ± 1.28 | 1.36 ± 1.00 | 0.5417 |
| RI1451 | 0.93 ± 0.31 | 0.85 ± 0.35 | 0.0604 |
| RI1451.7 | 1.15 ± 0.99 | 1.08 ± 1.14 | 0.5843 |
| RI1452.3 | 0.99 ± 0.35 | 0.97 ± 0.29 | 0.6581 |
| RI1456 | 1.07 ± 0.47 | 1.04 ± 0.47 | 0.5460 |
| RI1481.9 | 0.69 ± 0.91 | 0.62 ± 0.40 | 0.3467 |
| RI1492.9 | 1.09 ± 0.68 | 1.91 ± 7.46 | 0.1907 |
| RI1495 | 0.89 ± 0.42 | 0.90 ± 0.49 | 0.8107 |
| RI1497 | 1.09 ± 0.62 | 1.01 ± 0.55 | 0.2600 |
| RI1502.2 | 1.07 ± 0.60 | 1.18 ± 1.50 | 0.4131 |
| RI1512.1 | 0.71 ± 0.57 | 1.99 ± 11.29 | 0.1772 |
| RI1523.5 | 1.25 ± 2.48 | 1.02 ± 1.68 | 0.3492 |
| RI1524.7 | 0.58 ± 0.69 | 0.57 ± 0.29 | 0.9508 |
| RI1527.3 | 1.04 ± 0.65 | 1.14 ± 0.68 | 0.1993 |
| RI1535.3 | 0.76 ± 0.47 | 0.79 ± 0.44 | 0.5299 |
| RI1538.7 | 1.52 ± 2.28 | 1.79 ± 3.03 | 0.3910 |
| RI1543.1 | 1.04 ± 0.51 | 0.98 ± 0.41 | 0.2918 |
| RI1544.6 | 1.08 ± 0.56 | 1.08 ± 0.60 | 0.9884 |
| RI1550.7 | 1.58 ± 3.30 | 1.69 ± 3.24 | 0.7747 |
| RI1556.9 | 1.12 ± 0.72 | 1.24 ± 1.02 | 0.2601 |
| RI1557.6 | 1.18 ± 0.66 | 1.08 ± 0.47 | 0.1733 |
| RI1563 | 4.26 ± 7.62 | 4.61 ± 7.46 | 0.6955 |
| RI1572 | 0.73 ± 0.46 | 0.69 ± 0.35 | 0.3880 |
| RI1577.9 | 0.85 ± 0.88 | 0.93 ± 0.97 | 0.4460 |
| RI1586.3 | 1.06 ± 0.67 | 1.05 ± 0.58 | 0.8532 |
| RI1590.2 | 1.36 ± 0.56 | 1.42 ± 0.79 | 0.4231 |
| RI1605 | 1.28 ± 1.53 | 1.40 ± 1.69 | 0.5481 |
| RI1609 | 1.68 ± 9.59 | 1.79 ± 7.87 | 0.9159 |
| RI1612.1 | 0.94 ± 0.40 | 0.97 ± 0.44 | 0.6518 |
| RI1612.6 | 1.04 ± 0.71 | 1.10 ± 0.69 | 0.4991 |
| RI1615.8 | 1.54 ± 1.55 | 1.47 ± 1.23 | 0.6495 |
| RI1615.9 | 0.99 ± 0.44 | 0.98 ± 0.42 | 0.8128 |
| RI1620.8 | 0.79 ± 0.73 | 0.68 ± 0.49 | 0.1646 |
| RI1631.8 | 0.98 ± 0.37 | 0.98 ± 0.33 | 0.9277 |
| RI1637.1 | 0.78 ± 0.44 | 0.80 ± 0.47 | 0.6318 |
| RI1639.7 | 1.20 ± 0.97 | 1.18 ± 1.40 | 0.8419 |
| RI1646.7 | 1.46 ± 1.19 | 1.48 ± 1.21 | 0.8884 |
| RI1654.7 | 1.15 ± 3.08 | 1.14 ± 2.09 | 0.9723 |
| RI1665.4 | 2.92 ± 7.32 | 3.37 ± 7.59 | 0.6132 |
| RI1669.4 | 1.18 ± 0.80 | 1.12 ± 0.71 | 0.5185 |
| RI1686 | 1.17 ± 1.96 | 1.01 ± 0.41 | 0.3382 |
| RI1688.6 | 1.38 ± 1.84 | 1.37 ± 1.49 | 0.9819 |
| RI1691.7 | 0.75 ± 0.67 | 0.76 ± 0.60 | 0.8346 |
| RI1698.3 | 0.84 ± 1.69 | 0.73 ± 0.42 | 0.4622 |
| RI1704 | 1.08 ± 3.18 | 2.69 ± 8.35 | 0.0332 |
| RI1705.9 | 0.94 ± 0.46 | 0.96 ± 0.42 | 0.5975 |
| RI1707 | 1.08 ± 0.81 | 1.01 ± 0.48 | 0.3978 |
| RI1718.1 | 2.20 ± 3.68 | 2.76 ± 4.31 | 0.2357 |
| RI1726.6 | 1.56 ± 2.56 | 1.55 ± 2.21 | 0.9688 |
| RI1737.3 | 1.98 ± 4.66 | 2.46 ± 5.91 | 0.4415 |
| RI1741.1 | 1.22 ± 0.92 | 1.13 ± 0.82 | 0.3620 |
| RI1743 | 1.45 ± 1.20 | 1.93 ± 1.80 | 0.0081 |
| RI1746.5 | 1.49 ± 1.24 | 1.62 ± 1.66 | 0.4734 |
| RI1756.1 | 6.67 ± 21.52 | 12.33 ± 43.97 | 0.1684 |
| RI1758.2 | 1.20 ± 1.29 | 1.32 ± 1.95 | 0.5207 |
| RI1759.7 | 1.58 ± 1.61 | 1.63 ± 1.97 | 0.7958 |
| RI1786.5 | 1.57 ± 2.89 | 1.23 ± 0.98 | 0.1862 |
| RI1789.7 | 1.50 ± 2.59 | 1.45 ± 0.97 | 0.8605 |
| RI1795.2 | 1.00 ± 0.80 | 0.94 ± 0.60 | 0.4649 |
| RI1806.9 | 0.98 ± 0.62 | 0.98 ± 0.45 | 0.9596 |
| RI1809.1 | 1.46 ± 1.73 | 1.22 ± 0.80 | 0.1394 |
| RI1816 (anticipated: hexose) | 0.66 ± 0.60 | 0.56 ± 0.48 | 0.1484 |
| RI1816.6 | 2.01 ± 3.04 | 1.82 ± 2.83 | 0.5731 |
| RI1828.6 | 1.37 ± 1.22 | 1.39 ± 1.60 | 0.8835 |
| RI1833.7 | 1.28 ± 1.49 | 1.14 ± 1.28 | 0.4045 |
| RI1844.1 | 1.00 ± 0.80 | 0.96 ± 0.67 | 0.6350 |
| RI1858.2 | 0.94 ± 0.52 | 0.91 ± 0.46 | 0.5531 |
| RI1866.3 | 1.24 ± 2.19 | 1.27 ± 1.85 | 0.8892 |
| RI1884.1 | 1.70 ± 1.57 | 1.85 ± 2.66 | 0.5500 |
| RI1885.6 | 1.08 ± 0.59 | 1.07 ± 0.64 | 0.8747 |
| RI1888.8 | 0.96 ± 0.61 | 0.94 ± 0.82 | 0.8606 |
| RI1913.2 | 1.83 ± 2.30 | 1.80 ± 2.13 | 0.9166 |
| RI1951 | 1.23 ± 1.14 | 1.21 ± 0.86 | 0.8681 |
| RI1958.1 | 4.52 ± 7.26 | 2.95 ± 6.53 | 0.0553 |
| RI1965.5 | 3.22 ± 8.52 | 3.53 ± 10.26 | 0.7807 |
| RI1978.9 | 1.02 ± 0.93 | 1.08 ± 1.04 | 0.6439 |
| RI1984 | 1.19 ± 0.52 | 1.03 ± 0.46 | 0.0070 |
| RI1990.6 | 3.95 ± 21.98 | 13.10 ± 79.00 | 0.1838 |
| RI1999 | 1.70 ± 3.72 | 1.47 ± 3.13 | 0.5681 |
| RI2006 | 1.24 ± 0.51 | 1.45 ± 1.87 | 0.1962 |
| RI2006.5 | 0.92 ± 0.73 | 0.99 ± 0.68 | 0.3745 |
| RI2025.8 | 1.04 ± 0.57 | 0.96 ± 0.43 | 0.1820 |
| RI2035.8 | 1.50 ± 2.28 | 1.26 ± 1.69 | 0.3111 |
| RI2038.8 | 1.09 ± 0.79 | 1.07 ± 0.65 | 0.8440 |
| RI2043.1 | 1.18 ± 0.95 | 1.04 ± 0.53 | 0.1278 |
| RI2112.2 | 1.36 ± 0.96 | 1.29 ± 0.92 | 0.5848 |
| RI2115.6 | 1.04 ± 1.02 | 1.15 ± 0.82 | 0.3290 |
| RI2143 | 1.53 ± 1.65 | 1.31 ± 1.23 | 0.2077 |
| RI2144.7 | 1.02 ± 1.06 | 1.07 ± 0.84 | 0.6266 |
| RI2172.1 | 1.28 ± 1.50 | 1.27 ± 1.86 | 0.9890 |
| RI2174.4 | 0.64 ± 0.70 | 0.57 ± 0.38 | 0.2776 |
| RI2179.5 | 0.79 ± 1.23 | 0.72 ± 0.54 | 0.5490 |
| RI2184 | 1.25 ± 1.08 | 1.07 ± 0.93 | 0.1497 |
| RI2190.4 | 0.56 ± 0.84 | 0.67 ± 0.88 | 0.2829 |
| RI2202 | 1.06 ± 0.91 | 0.88 ± 0.52 | 0.0354 |
| RI2205.9 | 0.90 ± 0.89 | 0.86 ± 0.80 | 0.6699 |
| RI2211.9 | 1.36 ± 1.00 | 1.35 ± 0.98 | 0.9722 |
| RI2217.1 | 2.22 ± 6.25 | 1.43 ± 3.99 | 0.2034 |
| RI2219 | 1.00 ± 0.48 | 1.13 ± 0.46 | 0.0245 |
| RI2221.3 | 1.35 ± 1.58 | 1.52 ± 1.91 | 0.3987 |
| RI2230 | 1.11 ± 0.47 | 1.08 ± 0.51 | 0.6711 |
| RI2230.4 | 0.89 ± 0.76 | 0.80 ± 0.66 | 0.3115 |
| RI2237.1 | 1.11 ± 0.47 | 1.22 ± 0.59 | 0.0808 |
| RI2245 | 1.52 ± 2.41 | 1.81 ± 4.52 | 0.5003 |
| RI2246.8 | 1.07 ± 0.91 | 0.96 ± 0.70 | 0.2329 |
| RI2251.2 | 0.78 ± 0.40 | 0.78 ± 0.38 | 0.8741 |
| RI2259.5 | 1.72 ± 2.41 | 1.35 ± 1.61 | 0.1332 |
| RI2262.1 | 0.97 ± 1.03 | 1.10 ± 1.51 | 0.4132 |
| RI2262.9 | 1.00 ± 0.64 | 0.96 ± 0.60 | 0.6018 |
| RI2271.9 | 1.05 ± 0.61 | 1.04 ± 0.64 | 0.8422 |
| RI2282.8 | 1.07 ± 0.58 | 1.09 ± 0.63 | 0.8344 |
| RI2293.7 | 1.03 ± 0.62 | 0.94 ± 0.39 | 0.1415 |
| RI2316.5 | 1.32 ± 2.76 | 1.19 ± 1.21 | 0.6075 |
| RI2327.1 | 1.45 ± 1.34 | 1.24 ± 1.22 | 0.1596 |
| RI2330.5 | 0.97 ± 0.63 | 0.94 ± 0.66 | 0.7393 |
| RI2339.9 | 1.21 ± 1.12 | 1.03 ± 0.61 | 0.0851 |
| RI2348.6 | 1.26 ± 1.19 | 1.38 ± 1.23 | 0.3841 |
| RI2379.1 | 1.14 ± 0.48 | 1.13 ± 0.34 | 0.8528 |
| RI2406.3 | 1.13 ± 0.80 | 1.32 ± 1.54 | 0.1899 |
| RI2410.8 | 1.10 ± 1.13 | 1.06 ± 0.69 | 0.7300 |
| RI2414.8 | 0.92 ± 0.41 | 0.97 ± 0.39 | 0.2646 |
| RI2419.1 | 1.10 ± 1.08 | 1.21 ± 1.14 | 0.4149 |
| RI2427 | 2.08 ± 4.65 | 2.22 ± 4.95 | 0.8017 |
| RI2463 | 1.03 ± 0.75 | 1.07 ± 1.03 | 0.6660 |
| RI2464.9 | 1.00 ± 0.49 | 0.98 ± 0.42 | 0.6957 |
| RI2507.3 | 1.08 ± 0.46 | 1.07 ± 0.43 | 0.7410 |
| RI2509.6 | 1.11 ± 0.74 | 0.99 ± 0.54 | 0.1265 |
| RI2510 | 0.99 ± 0.32 | 0.95 ± 0.31 | 0.3004 |
| RI2510.7 | 1.25 ± 3.14 | 1.43 ± 3.31 | 0.6343 |
| RI2522.8 | 1.00 ± 0.47 | 0.89 ± 0.41 | 0.0436 |
| RI2537.7 | 1.17 ± 2.79 | 0.99 ± 0.39 | 0.4522 |
| RI2573.4 | 1.05 ± 0.61 | 1.00 ± 0.47 | 0.4426 |
| RI2600 | 2.37 ± 2.34 | 2.27 ± 2.19 | 0.7207 |
| RI2606.6 | 0.86 ± 1.58 | 1.48 ± 5.15 | 0.1702 |
| RI2681.4 | 1.28 ± 1.62 | 1.08 ± 1.15 | 0.2430 |
| RI2905.9 | 2.03 ± 3.82 | 1.58 ± 2.43 | 0.2400 |
| RI2970 | 1.01 ± 0.95 | 1.17 ± 0.92 | 0.1543 |
| RI2980 | 1.45 ± 1.99 | 1.05 ± 0.92 | 0.0298 |
| RI3134.6 | 0.91 ± 0.27 | 0.89 ± 0.27 | 0.4988 |
| RI3272 | 0.99 ± 0.28 | 0.97 ± 0.25 | 0.5300 |
| RI3286.5 | 1.14 ± 1.19 | 1.30 ± 1.58 | 0.3226 |
| RI3336.8 | 0.96 ± 0.49 | 1.00 ± 0.57 | 0.5153 |
| RI3353.8 | 1.13 ± 0.75 | 1.10 ± 0.69 | 0.7146 |
| RI3585.9 | 0.89 ± 0.36 | 0.85 ± 0.36 | 0.4342 |
| RI3683 | 0.78 ± 0.43 | 0.81 ± 0.42 | 0.5421 |
| RI3779.2 | 0.75 ± 0.22 | 0.75 ± 0.22 | 0.7082 |
| RI3808.1 | 1.61 ± 5.06 | 1.63 ± 3.76 | 0.9605 |
| RI3813.5 | 0.94 ± 0.40 | 0.93 ± 0.48 | 0.8412 |
| RI3988.6 | 0.95 ± 0.73 | 0.80 ± 0.69 | 0.0758 |
| RI4006.1 | 0.87 ± 0.46 | 0.83 ± 0.49 | 0.5004 |
| RI985.2 | 1.70 ± 2.11 | 1.24 ± 0.98 | 0.0181 |
| RI993.5 | 1.28 ± 1.15 | 1.30 ± 1.19 | 0.9063 |
| RI995.6 | 1.12 ± 0.83 | 1.00 ± 0.61 | 0.1663 |
| RI995.9 | 1.01 ± 0.30 | 1.04 ± 0.31 | 0.4141 |
| RI996.6 | 1.29 ± 1.63 | 1.44 ± 2.50 | 0.5663 |
