# Supplementary material for: Plasma Metabolome Signature Indicative of BRCA1 Germline Status Independent of Cancer Incidence
Source: Front Oncol. 2021 Apr 7;11:627217. doi: 10.3389/fonc.2021.627217 (PMC8058469; doi:10.3389/fonc.2021.627217)
Supplement: Supplementary file 2 [file Table_2.docx]

**Supplementary Material 2.** List of mean ± standard deviation of 78 metabolites with RSD <20%; p-values were calculated using a two-tailed Welch’s t-test. For metabolites identified via mass spectrometry metabolite libraries, metabolite names and derivatization details (MeOX: methoxyamine, TMS: trimethylsylyl) are specified. If metabolites had more than one peak, the main product is denoted as ‘MP’ and byproducts as ‘BP’.

| **metabolite** | **controls** | **g*BRCA1*+** | **p-value** |
| --- | --- | --- | --- |
| 1,5-anhydro-D-glucitol 4TMS | 0.92 ± 0.83 | 0.97 ± 0.49 | 0.5143 |
| 2-hydroxybutyric acid 2TMS | 1.26 ± 0.97 | 1.06 ± 0.76 | 0.0634 |
| 3-hydroxybutyric acid 2TMS | 1.41 ± 1.90 | 1.18 ± 1.45 | 0.2517 |
| aspartic acid 3TMS | 1.20 ± 0.54 | 1.17 ± 0.51 | 0.7385 |
| carbonic acid 1MeOX 2TMS | 0.97 ± 0.30 | 0.93 ± 0.27 | 0.2197 |
| cholesterol 1TMS | 0.84 ± 0.18 | 0.83 ± 0.17 | 0.6413 |
| citric acid 4TMS | 0.98 ± 0.28 | 1.03 ± 0.30 | 0.1754 |
| glucose 1MEOX 5TMS MP | 1.08 ± 0.19 | 1.07 ± 0.22 | 0.4865 |
| glucose 5TMS MP | 0.68 ± 0.35 | 0.69 ± 0.29 | 0.7549 |
| glutamic acid 3TMS | 0.95 ± 0.73 | 0.99 ± 0.59 | 0.6633 |
| glycerol 3TMS | 1.15 ± 0.41 | 1.24 ± 0.48 | 0.1001 |
| glycine 3TMS | 1.07 ± 0.38 | 1.15 ± 0.49 | 0.1030 |
| hexadecanoic acid 1TMS | 0.99 ± 0.44 | 0.96 ± 0.45 | 0.5438 |
| isoleucine 2TMS | 1.03 ± 0.48 | 1.06 ± 0.50 | 0.5596 |
| lactic acid 2TMS | 1.01 ± 0.43 | 0.94 ± 0.33 | 0.1513 |
| lysine 4TMS | 1.05 ± 0.55 | 1.04 ± 0.48 | 0.9449 |
| ornithine 4TMS | 1.06 ± 0.53 | 1.08 ± 0.57 | 0.8144 |
| oxalic acid 2TMS | 1.28 ± 3.52 | 1.06 ± 1.23 | 0.4829 |
| phenylalanine 2TMS | 1.00 ± 0.31 | 1.05 ± 0.33 | 0.1789 |
| phosphoric acid 3TMS | 0.94 ± 0.28 | 0.97 ± 0.25 | 0.3399 |
| pyroglutamic acid 2TMS | 1.08 ± 0.31 | 1.11 ± 0.39 | 0.5312 |
| pyruvic acid 1MEOX 1TMS | 1.00 ± 0.61 | 1.33 ± 0.89 | 0.0003 |
| serine 3TMS | 1.03 ± 0.37 | 1.06 ± 0.36 | 0.4742 |
| tryptophan 3TMS | 0.88 ± 0.35 | 0.90 ± 0.33 | 0.5478 |
| valine 2TMS | 1.00 ± 0.31 | 1.02 ± 0.31 | 0.4201 |
| RI1010.4 | 1.14 ± 0.55 | 1.10 ± 0.43 | 0.4770 |
| RI1020.1 | 0.99 ± 0.22 | 1.00 ± 0.22 | 0.8630 |
| RI1020.9 | 1.02 ± 0.23 | 1.02 ± 0.25 | 0.8172 |
| RI1030.2 | 1.11 ± 0.85 | 1.07 ± 0.43 | 0.6693 |
| RI1036.3 | 0.98 ± 0.21 | 0.99 ± 0.19 | 0.8716 |
| RI1040.8 | 1.00 ± 0.40 | 0.94 ± 0.32 | 0.1937 |
| RI1103.3 | 1.05 ± 0.39 | 1.07 ± 0.28 | 0.6818 |
| RI1112.6 | 1.05 ± 0.57 | 1.01 ± 0.24 | 0.4414 |
| RI1120.3 | 0.76 ± 0.37 | 0.75 ± 0.34 | 0.8514 |
| RI1140 | 0.95 ± 0.39 | 0.94 ± 0.31 | 0.6988 |
| RI1156.8 | 1.06 ± 0.35 | 1.06 ± 0.50 | 0.9153 |
| RI1258.9 | 1.09 ± 0.59 | 1.11 ± 0.60 | 0.7060 |
| RI1290.8 | 0.98 ± 0.36 | 0.98 ± 0.41 | 0.9211 |
| RI1301.5 | 2.86 ± 20.73 | 1.62 ± 6.65 | 0.4948 |
| RI1310.7 | 1.12 ± 0.72 | 1.06 ± 0.52 | 0.4165 |
| RI1313.6 | 1.05 ± 0.61 | 1.17 ± 0.50 | 0.0664 |
| RI1364.8 | 1.24 ± 1.19 | 1.12 ± 0.82 | 0.3393 |
| RI1376.6 | 1.03 ± 0.25 | 0.98 ± 0.19 | 0.0587 |
| RI1384.7 | 0.97 ± 0.38 | 0.93 ± 0.34 | 0.3544 |
| RI1389.4 | 0.96 ± 0.22 | 1.00 ± 0.25 | 0.1328 |
| RI1398.7 | 1.05 ± 0.48 | 1.11 ± 0.56 | 0.3235 |
| RI1451.7 | 1.15 ± 0.99 | 1.08 ± 1.14 | 0.5843 |
| RI1452.3 | 0.99 ± 0.35 | 0.97 ± 0.29 | 0.6581 |
| RI1456 | 1.07 ± 0.47 | 1.04 ± 0.47 | 0.5460 |
| RI1492.9 | 1.09 ± 0.68 | 1.91 ± 7.46 | 0.1907 |
| RI1535.3 | 0.76 ± 0.47 | 0.79 ± 0.44 | 0.5299 |
| RI1556.9 | 1.12 ± 0.72 | 1.24 ± 1.02 | 0.2601 |
| RI1557.6 | 1.18 ± 0.66 | 1.08 ± 0.47 | 0.1733 |
| RI1590.2 | 1.36 ± 0.56 | 1.42 ± 0.79 | 0.4231 |
| RI1615.9 | 0.99 ± 0.44 | 0.98 ± 0.42 | 0.8128 |
| RI1631.8 | 0.98 ± 0.37 | 0.98 ± 0.33 | 0.9277 |
| RI1705.9 | 0.94 ± 0.46 | 0.96 ± 0.42 | 0.5975 |
| RI1786.5 | 1.57 ± 2.89 | 1.23 ± 0.98 | 0.1862 |
| RI1885.6 | 1.08 ± 0.59 | 1.07 ± 0.64 | 0.8747 |
| RI1984 | 1.19 ± 0.52 | 1.03 ± 0.46 | 0.0070 |
| RI2006 | 1.24 ± 0.51 | 1.45 ± 1.87 | 0.1962 |
| RI2219 | 1.00 ± 0.48 | 1.13 ± 0.46 | 0.0245 |
| RI2230 | 1.11 ± 0.47 | 1.08 ± 0.51 | 0.6711 |
| RI2237.1 | 1.11 ± 0.47 | 1.22 ± 0.59 | 0.0808 |
| RI2251.2 | 0.78 ± 0.40 | 0.78 ± 0.38 | 0.8741 |
| RI2271.9 | 1.05 ± 0.61 | 1.04 ± 0.64 | 0.8422 |
| RI2282.8 | 1.07 ± 0.58 | 1.09 ± 0.63 | 0.8344 |
| RI2293.7 | 1.03 ± 0.62 | 0.94 ± 0.39 | 0.1415 |
| RI2379.1 | 1.14 ± 0.48 | 1.13 ± 0.34 | 0.8528 |
| RI2507.3 | 1.08 ± 0.46 | 1.07 ± 0.43 | 0.7410 |
| RI2510 | 0.99 ± 0.32 | 0.95 ± 0.31 | 0.3004 |
| RI2510.7 | 1.25 ± 3.14 | 1.43 ± 3.31 | 0.6343 |
| RI3134.6 | 0.91 ± 0.27 | 0.89 ± 0.27 | 0.4988 |
| RI3272 | 0.99 ± 0.28 | 0.97 ± 0.25 | 0.5300 |
| RI3585.9 | 0.89 ± 0.36 | 0.85 ± 0.36 | 0.4342 |
| RI3779.2 | 0.75 ± 0.22 | 0.75 ± 0.22 | 0.7082 |
| RI3813.5 | 0.94 ± 0.40 | 0.93 ± 0.48 | 0.8412 |
| RI995.9 | 1.01 ± 0.30 | 1.04 ± 0.31 | 0.4141 |
