# Supplementary material for: Plasma Metabolome Signature Indicative of BRCA1 Germline Status Independent of Cancer Incidence
Source: Front Oncol. 2021 Apr 7;11:627217. doi: 10.3389/fonc.2021.627217 (PMC8058469; doi:10.3389/fonc.2021.627217)
Supplement: Supplementary file 3 [file Table_3.docx]

**Supplementary Material 3.** Overview of all classifiers and their performance regarding machine learning sub-analyses.

|  | raw | averaged | averaged RSD<20 | ratios | ratios+averaged RSD<20 |
| --- | --- | --- | --- | --- | --- |
| **iterations LogitBoost** | 4 | 5 | 4 | 13 | 13 |
| **10x cross**  **validation** | 60,58 | 56.9444 | 60.4167 | 63.8889 | 64.5833 |
| **test set all** | 67.7083 | 68.75 | 63.1944 | 81.9444 | 81.9444 |
| **G1** | 61.5385 | 60 | 58.4615 | 80 | 80 |
| **G2** | 75.4386 | 78.9474 | 75.4386 | 84.2105 | 84.2105 |
| **G3** | 65.9091 | 68.1818 | 45.4545 | 81.8182 | 81.8182 |
| **classifier** | Class BRCA1 :  0.34 +  [RI1984] * -0.32 +  [lactic_acid_2TMS] * -0.56 +  [pyruvic_acid_1MEOX_1TMS] * 0.48 | Class BRCA1 :  0.16 +  [RI1704] * 0.05 +  [RI1743] * 0.18 +  [RI1984] * -0.58 +  [RI3272] * -0.19 +  [pyruvic_acid_1MEOX_1TMS] * 0.3 | Class BRCA1 :  0.07 +  [RI1984] * -0.58 +  [citric_acid_4TMS] * 0.68 +  [lactic_acid_2TMS] * -0.48 +  [pyruvic_acid_1MEOX_1TMS] * 0.3 | Class BRCA1 :  0.22 +  [RI1020.9:RI1631.8] * -0.84 +  [RI1103.3:glucose_1MEOX_5TMS_MP] * 1.41 +  [RI1140:RI1364.8] * 1.1 +  [RI1313.6:2-hydroxybutyric_acid_2TMS] * 0.53 +  [RI1313.6:lactic_acid_2TMS] * 1.11 +  [RI1451.7:RI1705.9] * -0.73 +  [RI1452.3:carbonic_acid_1MeOX_2TMS] * 2.74 +  [RI1984:citric_acid_4TMS] * -1.13 +  [RI1984:pyruvic_acid_1MEOX_1TMS] * -1.21 +  [RI2219:RI2230] * 0.92 +  [RI2293.7:glucose_5TMS_MP] * -0.83 +  [RI2510.7:RI3813.5] * 0.62 +  [lactic_acid_2TMS:pyruvic_acid_1MEOX_1TMS] * -0.87 | Class BRCA1 :  0.22 +  [RI1020.9:RI1631.8] * -0.84 +  [RI1103.3:glucose_1MEOX_5TMS_MP] * 1.41 +  [RI1140:RI1364.8] * 1.1 +  [RI1313.6:2-hydroxybutyric_acid_2TMS] * 0.53 +  [RI1313.6:lactic_acid_2TMS] * 1.11 +  [RI1451.7:RI1705.9] * -0.73 +  [RI1452.3:carbonic_acid_1MeOX_2TMS] * 2.74 +  [RI1984:citric_acid_4TMS] * -1.13 +  [RI1984:pyruvic_acid_1MEOX_1TMS] * -1.21 +  [RI2219:RI2230] * 0.92 +  [RI2293.7:glucose_5TMS_MP] * -0.83 +  [RI2510.7:RI3813.5] * 0.62 +  [lactic_acid_2TMS:pyruvic_acid_1MEOX_1TMS] * -0.87 |
